# Sensorless Coupling Estimation via the Input Reflection Coefficient of a Chireix Combiner for Adaptive Wearable Wireless Power Transfer

**DOI:** 10.3390/s26185818

**Published:** 2026-09-14

**Authors:** Nizar Brahim, Giuseppina Monti, Luciano Tarricone, Hichem Taghouti

**Affiliations:** 1 LAPER Laboratory, Faculty of Sciences of Tunis (FST), El Manar University, Tunis 1068, Tunisia; 2 Department of Innovation Engineering, University of Salento, 73100 Lecce, Italy; giuseppina.monti@unisalento.it (G.M.); luciano.tarricone@unisalento.it (L.T.); 3 High Institute of Environmental Sciences and Technologies of Borj Cedria, University of Carthage, Borj Cedria 2084, Tunisia; hichem.taghouti@fst.utm.tn

**Keywords:** wireless power transfer, Chireix combiner, coupling estimation, reflection phase, RF sensing, wearable systems

## Abstract

Adaptive wireless power transfer (WPT) systems for wearable and biomedical applications require continuous knowledge of the magnetic coupling coefficient to maintain stable power delivery under motion-induced variations. Conventional approaches rely on auxiliary sensors or dedicated RF measurement stages, which increase system complexity and limit integration in compact platforms. This paper introduces a sensorless coupling estimation method based on the input reflection phase of a Chireix outphasing combiner operating in in-phase mode (φ=0°). The reflection phase $∠\Gamma_{in}$ exhibits a numerically monotonic dependence on the coupling coefficient over the wearable-relevant range k∈[0.05,0.70], arising from a structural decoupling between coupling-dependent conductance and fixed susceptive compensation in the combiner input admittance. The method enables coupling estimation using only intrinsic RF observables at the transmitter port, without interrupting power transfer or requiring additional sensing hardware, via a two-step protocol combining sub-millisecond sensing with coupling-aware outphasing control. Validation combines analytical modeling, numerical evaluation, Monte Carlo analysis under component tolerances, and circuit-level S-parameter simulation at 13.56 MHz, demonstrating robust monotonic behavior under component variations, phase noise, and resonant detuning. The framework reinterprets the Chireix combiner as a dual-function structure enabling simultaneous power combining and coupling observability, providing a low-overhead solution for adaptive wearable WPT systems.

## 1. Introduction

Wireless power transfer (WPT) has become a key enabling technology for wearable and biomedical electronic systems requiring continuous energy delivery without physical connectors. Applications such as physiological monitoring, motion tracking, and implantable devices demand compact, efficient, and adaptive power delivery architectures capable of maintaining stable operation under dynamic changes in coil alignment, posture variation, and tissue loading conditions [1,2,3,4,5]. In particular, the 13.56 MHz industrial, scientific, and medical (ISM) band remains widely adopted due to its favorable compromise among coil miniaturization, near-field magnetic coupling efficiency, and regulatory compatibility [6,7].

Recent comprehensive reviews have highlighted the evolution of WPT system design, emphasizing the importance of coupling-aware control strategies and adaptive compensation network optimization [8]. Modern approaches address misalignment robustness through dynamic coil reconfiguration [9] and multi-frequency operation to broaden the efficiency envelope [10], while flexible winding structures have recently been proposed to enhance coupling performance under mechanical deformation, which is relevant to wearable and on-body platforms [11].

A fundamental challenge in resonant inductive WPT systems is the strong dependence of transferred power and impedance matching conditions on the magnetic coupling coefficient *k*. Since *k* varies continuously with distance, orientation, and biological motion, adaptive control strategies require real-time or near-real-time knowledge of the coupling state to maintain optimal performance [12,13]. Without such information, systems must either operate conservatively, sacrificing efficiency, or rely on frequent recalibration, both of which are unsuitable for autonomous wearable operation. Existing coupling estimation techniques typically rely on indirect electrical observables such as S-parameters, input impedance tracking, reflected power measurements, or transmission coefficient monitoring [14,15]. While effective in controlled environments, S-parameter-based approaches require vector network analyzers or dedicated RF measurement infrastructure and are therefore incompatible with fully embedded systems. Impedance tracking methods reduce hardware requirements but often require dedicated probing phases that interrupt normal power transfer. Furthermore, both approaches can suffer from reduced accuracy under detuning conditions introduced by the human body [16].

To overcome these limitations, recent research has explored sensor-augmented and self-tuning WPT architectures. Sensor-based approaches, including magnetic field sensors, capacitive proximity detectors, and received signal strength indicators (RSSI), improve observability but increase system complexity, calibration requirements, and sensitivity to environmental variability [16,17]. Self-tuning WPT systems based on impedance matching networks and load-independent operating regimes improve robustness but typically do not provide an explicit estimate of k, which remains essential for predictive control and system-level optimization [18].

In parallel, RF power amplifier architectures such as outphasing and Doherty systems have demonstrated that load-dependent impedance transformations can be exploited for efficiency optimization and adaptive control. Chireix outphasing combiners introduce controlled reactive compensation that shapes the load trajectory seen by the power amplifiers, enabling high-efficiency operation over a range of load conditions [19,20]. However, these structures have traditionally been analyzed exclusively from the perspective of power combining and efficiency enhancement, while their input impedance behavior has not been investigated as a source of information about the coupled WPT system. This paper shows that, under in-phase excitation (φ=0°), the Chireix combiner exhibits intrinsic coupling observability, whereby the magnetic coupling state is encoded in the input reflection characteristics of the transmitter. Specifically, the input reflection phase becomes a numerically monotonic function of the magnetic coupling coefficient, enabling the coupling state to be inferred directly from an externally measurable RF quantity available at the transmitter input. This behavior arises from a structural separation of the input admittance under ideal resonant conditions, in which the coupling-dependent conductance and the fixed Chireix compensation susceptance jointly produce a coupling-sensitive impedance transformation. To the best of the authors’ knowledge, the interpretation of a Chireix outphasing combiner as an intrinsic RF sensing interface for estimating the coupling state of a resonant inductive WPT link has not been previously investigated in the context of adaptive wearable applications. This perspective differs from prior reflection- or impedance-based coupling–sensing approaches, which have primarily considered conventional WPT transmitter architectures rather than the intrinsic input-impedance behavior of an outphasing combiner.

The recognition of this intrinsic coupling observability enables a sensorless coupling estimation framework requiring no dedicated sensing hardware beyond standard RF front-end components. Based on this principle, a two-step operating protocol is developed, consisting of a short sensing interval followed by coupling-aware outphasing control for adaptive power delivery. The proposed framework is evaluated through a progressive verification strategy addressing the existence, sensitivity, robustness, and practical measurement limitations of the coupling-dependent phase observable. Complementary analytical derivation, numerical evaluation, statistical robustness analysis, and independent circuit-level S-parameter simulations at 13.56 MHz are employed to establish the physical basis of the sensing mechanism and assess its feasibility under practical non-idealities.

The remainder of this paper is organized as follows. Section 2 reviews related work. Section 3 presents the system model. Section 4 derives the sensing principle. Section 5 introduces the estimation protocol. Section 6 presents the numerical evaluation, robustness analysis, and independent circuit-level verification of the proposed coupling observability framework. Section 7 discusses implementation aspects, practical considerations, and limitations. Finally, Section 8 concludes the paper.

## 2. Related Work

### 2.1. Coupling Estimation in Inductive Wireless Power Transfer

Magnetic coupling estimation is a central requirement in adaptive WPT systems, since k directly governs impedance transformation, delivered power, and end-to-end efficiency [18]. Classical approaches infer k indirectly through measurable electrical quantities such as input impedance, reflected power, or transmission scattering parameters. S-parameter-based methods (e.g., $S_{21}$ tracking) have been widely used in resonant inductive links to estimate coil separation or misalignment [14,21], but they require vector network analyzers or dedicated RF measurement chains, making them unsuitable for fully embedded wearable operation.

Alternative impedance-based tracking methods estimate coupling by monitoring variations in input resistance or resonant frequency shift of the transmitter coil [15,18]. While these techniques require less hardware, they still need either interruption of normal power transfer or dedicated excitation signals. Furthermore, their accuracy degrades under reactive loading variations and detuning effects introduced by biological tissue environments [18].

More recently, machine learning and advanced signal-processing approaches have emerged as alternatives to traditional methods. Machine learning-based parameter identification directly identifies the coupling coefficient from transmitter-side voltage and current measurements [22,23,24], thereby eliminating the need for dedicated measurement phases or additional sensing hardware.

### 2.2. Sensor-Augmented Approaches for Distance and Coupling Monitoring

Several works have explored explicit sensing modalities [16,17]. Magnetic field sensing using Hall-effect devices provides direct measurement of field strength variation with distance, but it requires additional sensors, magnetic biasing elements, and careful spatial alignment. Capacitive proximity sensing has been investigated for short-range distance estimation, but its performance is strongly influenced by environmental permittivity variations, making it less robust in dynamic on-body conditions. RSSI-based methods are simple to implement but suffer from nonlinearity, multipath sensitivity, and calibration drift in near-field inductive regimes. As a result, sensor-augmented approaches generally improve observability at the cost of increased hardware complexity and reduced robustness.

### 2.3. Self-Tuning and Physics-Assisted Adaptive WPT Systems

Recent research has shifted toward self-adaptive WPT architectures exploiting intrinsic circuit physics. Techniques based on impedance matching networks, load-independent operating regimes, and resonant frequency tuning have demonstrated improved robustness against coupling variation [18]. Advanced concepts such as parity–time symmetric WPT systems and exceptional-point-based enhancement mechanisms have been proposed to increase sensitivity or stabilize transfer efficiency [25].

Contemporary advances leverage real-time parameter identification for dynamic optimization under environmental variation. Energy injection strategies using online mutual inductance and load identification enable adaptive voltage and phase control for optimal matching [26], while parity–time symmetric operation at exceptional points provides automatic frequency adjustment without explicit coupling measurement [25]. Classical self-tuning approaches based on scattering parameter measurement and two-sided power matching provide a foundational framework for achieving resonant conditions under dynamic coil motion [27].

### 2.4. RF-Based Impedance Sensing in Power Electronics

In outphasing and Doherty power amplifiers, load-dependent impedance transformations have been studied extensively for efficiency optimization [19,20]. Chireix outphasing combiners exhibit strong dependence of internal node impedance on load conditions and phase excitation, making them potential candidates for sensing-oriented interpretation. Recent work has extended this theoretical foundation through generalized Doherty–Chireix continuum formulations aimed at bandwidth enhancement [28], and broader surveys have reviewed design strategies for broadband and multi-band outphasing power amplifiers [29]. However, existing works primarily treat the combiner as a power-combining structure. The observable quantities used in prior studies are typically output power, efficiency, or harmonic content, rather than phase-resolved reflection characteristics at the input port. Consequently, the intrinsic sensing potential of the combiner’s input reflection coefficient has remained largely unexplored in the context of WPT coupling estimation.

### 2.5. Positioning and Gap

From the above literature, three main limitations can be identified: (1) hardware dependency—most coupling estimation methods require additional sensors or external RF instrumentation [16,17]; (2) operational interruption—many impedance- or S-parameter-based techniques require dedicated measurement phases that perturb normal power delivery [14,15]; and (3) lack of physically direct sensing observables—existing self-tuning methods typically optimize efficiency but do not provide a directly measurable, monotonic, and invertible physical quantity linked to coupling [18,25].

This work introduces a sensorless estimation framework that exploits the input reflection phase of a Chireix outphasing combiner as an intrinsic sensing observable, enabling real-time, physically grounded, and fully embedded coupling estimation suitable for wearable WPT systems.

## 3. System Model

### 3.1. Outphasing WPT Transmitter Architecture

The considered system consists of a dual-branch Chireix outphasing transmitter driving a resonant inductive WPT link at 13.56 MHz [6]. Two identical PA branches deliver constant-envelope signals phase-shifted by ±φ/2, with the outphasing angle φ controlling output power through vector summation. In practical outphasing transmitters, each branch is commonly realized using high-efficiency switching-mode amplifier classes such as Class-E [30], following established RF power amplifier design principles [31]. The branch outputs are recombined by a Chireix combining network with shunt susceptance compensation elements $\pm jB_{c}$, following classical outphasing theory [19] extended in [20,32] for modern RF implementations.

**Figure 1:**
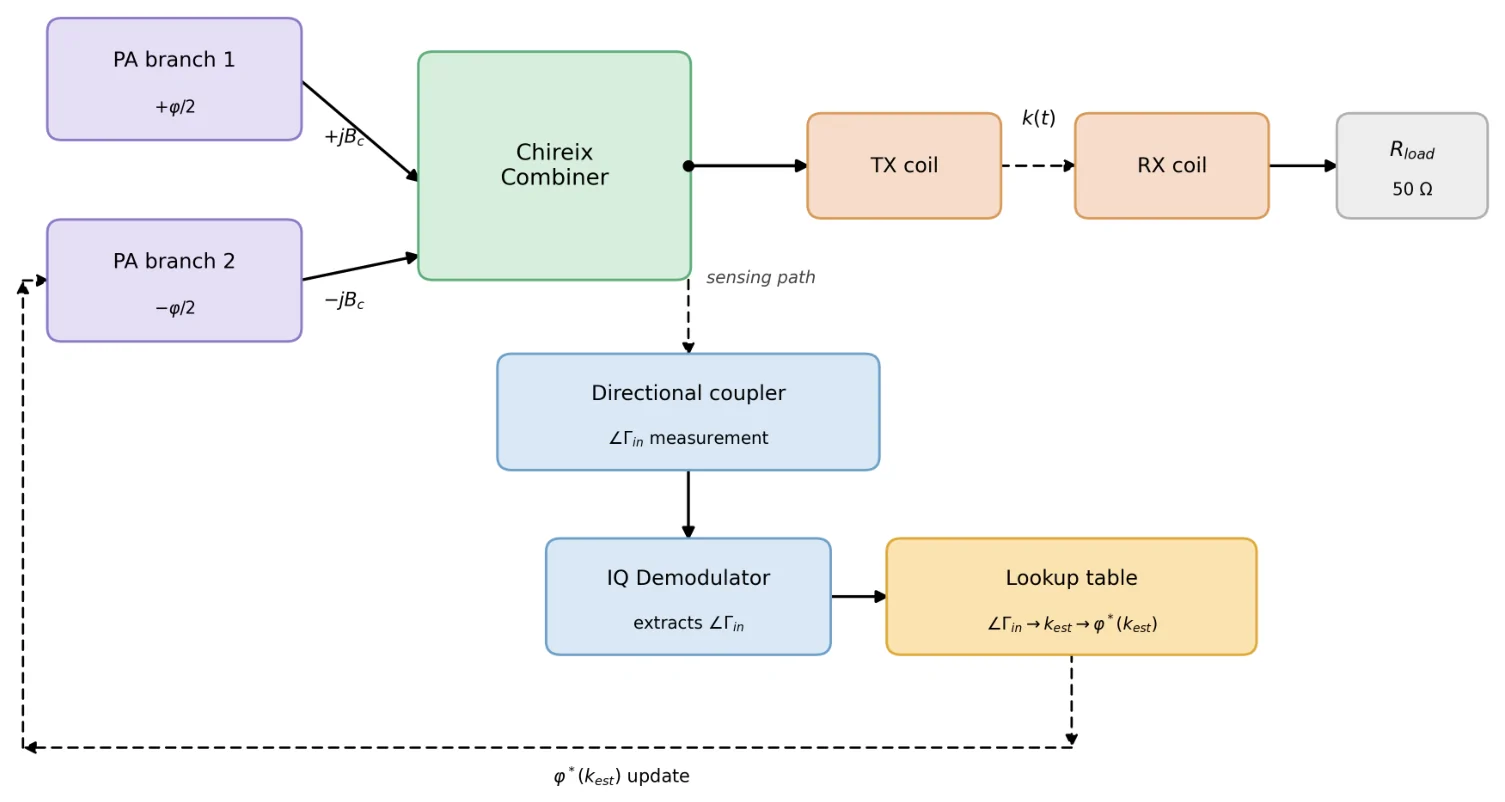
System architecture of the Chireix outphasing WPT transmitter with sensorless coupling estimation. Two PA branches deliver constant-envelope signals phase-shifted by ±φ/2 and recombined through the Chireix combining network with shunt compensation elements $\pm jB_{c}$. At φ=0°, the input reflection phase $∠\Gamma_{\mathrm{in}}$ is measured via a directional coupler and IQ demodulator, mapped to the coupling estimate $k_{\mathrm{est}}$ through a precomputed lookup table, and used to update the optimal outphasing angle $\varphi^{*}\left(k_{\mathrm{est}}\right)$ for adaptive power delivery.

The total end-to-end efficiency is:

(1) $$\eta_{\mathrm{total}}\left(\varphi ,k\right)=\eta_{\mathrm{PA}}\cdot \eta_{\mathrm{combiner}}\left(\varphi ,k\right)\cdot \eta_{\mathrm{WPT}}\left(k\right)$$

where $\eta_{\mathrm{PA}}$ is the constant PA branch efficiency, $\eta_{\mathrm{combiner}}$ captures combining losses, and $\eta_{\mathrm{WPT}}$ is the wireless link efficiency. The adaptive control objective is to select $\varphi^{*}\left(k\right)$ that maximizes $\eta_{\mathrm{total}}$ for each instantaneous coupling condition, following the coupling-aware framework of [33]. Figure 1 illustrates the system architecture.

### 3.2. Inductive WPT Link Model

The inductive WPT link is modeled as two magnetically coupled resonators series-tuned to 13.56MHz [6,7,34]. The mutual inductance is $M=k\sqrt{L_{\mathrm{tx}}\cdot L_{\mathrm{rx}}}$, where $L_{\mathrm{tx}}=L_{\mathrm{rx}}=1.17\;\mu H$. Under resonant conditions, the reflected impedance at the transmitter port is purely resistive:

(2) $$Z_{\mathrm{refl}}\left(k\right)=\frac{{\left(\omega M\right)}^{2}}{R_{\mathrm{rx}}}=\frac{\left(\omega^{2}k^{2}L_{\mathrm{tx}}L_{\mathrm{rx}}\right)}{R_{\mathrm{rx}}}=199k^{2}\;\left[\Omega \right]$$

with $R_{\mathrm{rx}}=R_{\mathrm{load}}=50\;\Omega$. As a numerical check: $Z_{\mathrm{refl}}\left(0.1\right)=1.99\;\Omega$, $Z_{\mathrm{refl}}\left(0.5\right)=49.75\;\Omega$, and $Z_{\mathrm{refl}}\left(0.9\right)=161.0\;\Omega$. The effective load seen by the Chireix combiner is:

(3) $$Z_{\mathrm{eff}}\left(k\right)=R_{\mathrm{tx}}+Z_{\mathrm{refl}}\left(k\right)=1+199k^{2}\;\left[\Omega \right]$$

where $R_{\mathrm{tx}}=1\;\Omega$ is the transmitter coil series resistance. $Z_{\mathrm{eff}}\left(k\right)$ increases strictly with *k*, spanning from 1.50Ω at k=0.05 to 98.51Ω at k=0.70.

The impedance model $Z_{\mathrm{eff}}\left(k\right)=1+199k^{2}$ represents a fundamental relationship in series-tuned resonant inductive WPT systems and generalizes beyond the specific coil parameters chosen here. The model structure arises from the coupling-dependent mutual inductance $M=k\sqrt{L_{\mathrm{tx}}\cdot L_{\mathrm{rx}}}$ and the load resistance $R_{x}=50\;\Omega$, which are standard design choices in practical WPT systems. The numerical coefficient 199 is derived from the operating frequency (13.56 MHz) and coil inductance values ($L_{\mathrm{tx}}=L_{\mathrm{rx}}=1.17\;\mu H$); however, the fundamental $k^{2}$ dependence of the reflected impedance is universal for all series-tuned resonant configurations, independent of these specific parameter values. Consequently, variations in coil size, frequency, or load impedance would modify only the numerical coefficient, not the monotonic $k^{2}$ relationship itself. This generality ensures that the phase–coupling mapping derived in Section 4, while numerically calibrated for the 13.56 MHz wearable WPT configuration, reflects a structural property of resonant inductive systems that remains valid across a range of practical WPT implementations. Extension to alternative parameter sets (different frequencies, coil geometries, or load values) would require recalibration of the lookup table but would preserve the underlying monotonic phase–coupling behavior demonstrated in this work.

### 3.3. Chireix Combiner Input Admittance

For a Chireix combiner with compensation susceptance $B_{c}=\tan\left(\varphi_{c}\right)/Z_{0}=\tan\left(70{}^{\circ}\right)/50=0.05495\;S$, the input admittance of each branch under outphasing excitation at angle φ is:

(4) $$Y_{\mathrm{branch}}\left(\varphi ,k\right)=\left(\frac{1}{Z_{\mathrm{eff}}}\right)\cos^{2}\left(\varphi /2\right)+j\left[\left(\frac{1}{Z_{\mathrm{eff}}}\right)\sin\left(\varphi /2\right)\cos\left(\varphi /2\right)-B_{c}\right]$$

The total input admittance, summing both branches symmetrically, is:

(5) $$Y_{\mathrm{in}}\left(\varphi ,k\right)=2\left(\frac{1}{Z_{\mathrm{eff}}}\right)\cos^{2}\left(\varphi /2\right)-j2\left[\left(\frac{1}{Z_{\mathrm{eff}}}\right)\sin\left(\varphi /2\right)\cos\left(\varphi /2\right)-B_{c}\right]$$

This expression forms the foundation for the sensing principle developed in Section 4.

### 3.4. Practical Non-Idealities in the Outphasing Transmitter

In practical Chireix implementations, additional non-idealities arise from PA branch asymmetry, including gain imbalance, finite phase skew, and nonlinear AM–PM conversion. These effects prevent perfect cancellation of reactive components at φ=0°, resulting in a residual complex offset in the input admittance. Importantly, these imperfections do not alter the underlying coupling-dependent modulation mechanism, but they introduce a slowly varying bias term in the phase response $∠\Gamma_{\mathrm{in}}\left(k\right)$.

As a result, the sensing function should be interpreted as a perturbed monotonic mapping rather than an exact analytical relation. The robustness observed in Section 6 is therefore attributed to structural dependence on impedance modulation rather than ideal cancellation.

### 3.5. System Parameters

Table 1 summarizes the system parameters used throughout the analytical derivation and numerical evaluation in this paper.

## 4. Analytical Derivation of the Sensing Principle

### 4.1. Analytical Decoupling at φ=0° Under Ideal Assumptions

The sensing principle emerges from evaluating Equation (5) at φ=0°. Setting φ=0° gives cos(0)=1 and sin(0)=0, yielding:

(6) $$Y_{\mathrm{in}}\left(0,k\right)=\frac{2}{Z_{\mathrm{eff}}\left(k\right)}-j2B_{c}$$

This expression has a critical structural consequence under ideal resonant assumptions: the imaginary part of $Y_{\mathrm{in}}$ is identically equal to $-2B_{c}=-0.10990\;S$, a constant determined solely by the combiner design parameter $B_{c}$, independent of *k*. The real part, $2/Z_{\mathrm{eff}}\left(k\right)$, varies strictly with *k* through Equation (3). Numerical verification confirms that Im(Yin) equals $-2B_{c}$ to eight significant figures across the full coupling range. This constancy is an exact consequence of the φ=0° operating condition under the assumed ideal model.

### 4.2. Input Reflection Coefficient as Sensing Observable

The corresponding input impedance at φ=0° is:

(7) $$Z_{\mathrm{in}}\left(0,k\right)=\frac{Z_{\mathrm{eff}}\left(k\right)}{\left[2-j2B_{c}\cdot Z_{\mathrm{eff}}\left(k\right)\right]}$$

The input reflection coefficient referred to $Z_{0}=50\;\Omega$ is:

(8) $$\Gamma_{\mathrm{in}}\left(k\right)=\frac{\left[Z_{\mathrm{in}}\left(0,k\right)-Z_{0}\right]}{\left[Z_{\mathrm{in}}\left(0,k\right)+Z_{0}\right]}$$

Substituting Equation (7) and rearranging gives:

(9) $$\Gamma_{\mathrm{in}}\left(k\right)=\frac{\left[Z_{\mathrm{eff}}\left(1+j100B_{c}\right)-100\right]}{\left[Z_{\mathrm{eff}}\left(1-j100B_{c}\right)+100\right]}$$

where $Z_{\mathrm{eff}}=1+199k^{2}$ from Equation (3). The phase, which serves as the sensing observable, is:

(10) $$∠\Gamma_{\mathrm{in}}\left(k\right)=\arg\left\{Z_{\mathrm{eff}}\left(1+j100B_{c}\right)-100\right\}-\arg\left\{Z_{\mathrm{eff}}\left(1-j100B_{c}\right)+100\right\}$$

### 4.3. Numerical Verification of Monotonicity

Equation (10) is evaluated numerically over 2000 uniformly spaced points spanning k∈[0.05,0.70]. The derivative $\frac{d(∠\Gamma_{\mathrm{in}})}{dk}$, computed using central differences, satisfies:

(11) $$\frac{d(∠\Gamma_{\mathrm{in}})}{dk}\in \left[-71.20,-3.56\right]\;\deg/\mathrm{unit}\left[k\right]\;\mathrm{over}\;k\in \left[0.05,0.70\right]$$

The derivative is negative throughout the evaluated range, with no sign changes observed across all 2000 evaluation points. The phase $∠\Gamma_{\mathrm{in}}\left(k\right)$ is therefore numerically monotonic (strictly decreasing) over the validated range, with:

(12) $$∠\Gamma_{\mathrm{in}}\left(k=0.05\right)=179.86{}^{\circ},\;∠\Gamma_{\mathrm{in}}\left(k=0.70\right)=160.02{}^{\circ},\;\mathrm{Total}\;\mathrm{variation}:\;19.84{}^{\circ}$$

The effectiveness of the sensing mechanism is governed by the local sensitivity $\left|\frac{d(∠\Gamma_{\mathrm{in}})}{dk}\right|$, which directly determines the coupling estimation resolution. The phase–coupling mapping exhibits a pronounced sensitivity peak in the weak-to-moderate coupling regime, corresponding to the most relevant operational range in wearable WPT scenarios. The peak sensitivity of 71.20deg/unit[k] occurs at k=0.258. At k=0.1 and k=0.5, the sensitivity is 14.20deg/unit[k] and 15.81deg/unit[k], respectively. This numerically verified behavior supports a locally injective mapping within the validated range under the assumptions of the lumped-element resonant model.

### 4.4. Structural Observability: The Impedance Separability Condition

The monotonic phase–coupling mapping is not a generic property of resonant inductive WPT systems. In conventional single-port WPT architectures, variations in coupling simultaneously perturb both resistive and reactive components of the input impedance in a coupled manner, preventing isolation of a single monotonic observable.

The Chireix architecture, by contrast, introduces a controlled dual-branch interference mechanism under φ=0° that yields an input admittance decomposable as follows:

(13) $$Y_{\mathrm{in}}\left(0,k\right)=G\left(k\right)+jB$$

where:

(14) $$G\left(k\right)=\frac{2}{Z_{\mathrm{eff}}\left(k\right)}$$

is a monotonically increasing function of *k*, and $B=-2B_{c}$ is a constant determined solely by the combiner design. This impedance separability condition isolates the coupling-dependent information into a single real component, enabling unambiguous phase encoding of *k* in the reflection coefficient.

An outphasing system satisfying this separability condition admits intrinsic coupling observability: variations in magnetic coupling are mapped onto a single observable degree of freedom—the phase of $\Gamma_{\mathrm{in}}$—without requiring auxiliary sensing hardware. This structural property is a consequence of the Chireix outphasing topology and is generally absent in conventional single-branch resonant WPT transmitters. Physical Intuition: The impedance separability at φ=0° can be understood as follows. In conventional single-branch WPT transmitters, coupling variations simultaneously perturb both the resistive ($R_{\mathrm{eq}}$) and reactive ($X_{\mathrm{eq}}$) components of the equivalent load impedance in a coupled manner. This coupling of real and imaginary parts means that no single electrical observable cleanly isolates the coupling state—the impedance angle and magnitude both change with *k*, making inversion ambiguous. The Chireix outphasing architecture breaks this coupling through a dual-branch interference mechanism: the two PA branches, when driven in phase (φ=0°), create a special symmetry wherein the reactive components from both branches combine constructively with the fixed compensation susceptance $B_{c}$, resulting in a total susceptance that is independent of *k*. This leaves only the resistance to vary with coupling, enabling a clean one-to-one mapping from impedance (or reflection coefficient phase) to *k*. This separability is not accidental; it is a direct consequence of the Chireix topology and does not occur in conventional transmitters, explaining why sensorless coupling observability has not been reported previously for single-branch WPT systems.

The validity of this observability condition is restricted to the following: (1) near-resonant operation, where the reflected impedance is predominantly resistive; (2) continuous and strictly increasing $Z_{\mathrm{eff}}\left(k^{2}\right)$ within the operational range; and (3) in-phase excitation (φ=0°) that minimizes reactive power circulation. Outside these conditions, monotonicity must be re-evaluated using system-specific modeling.

### 4.5. Sensitivity to Compensation Angle $\varphi_{c}$

**Figure 2:**
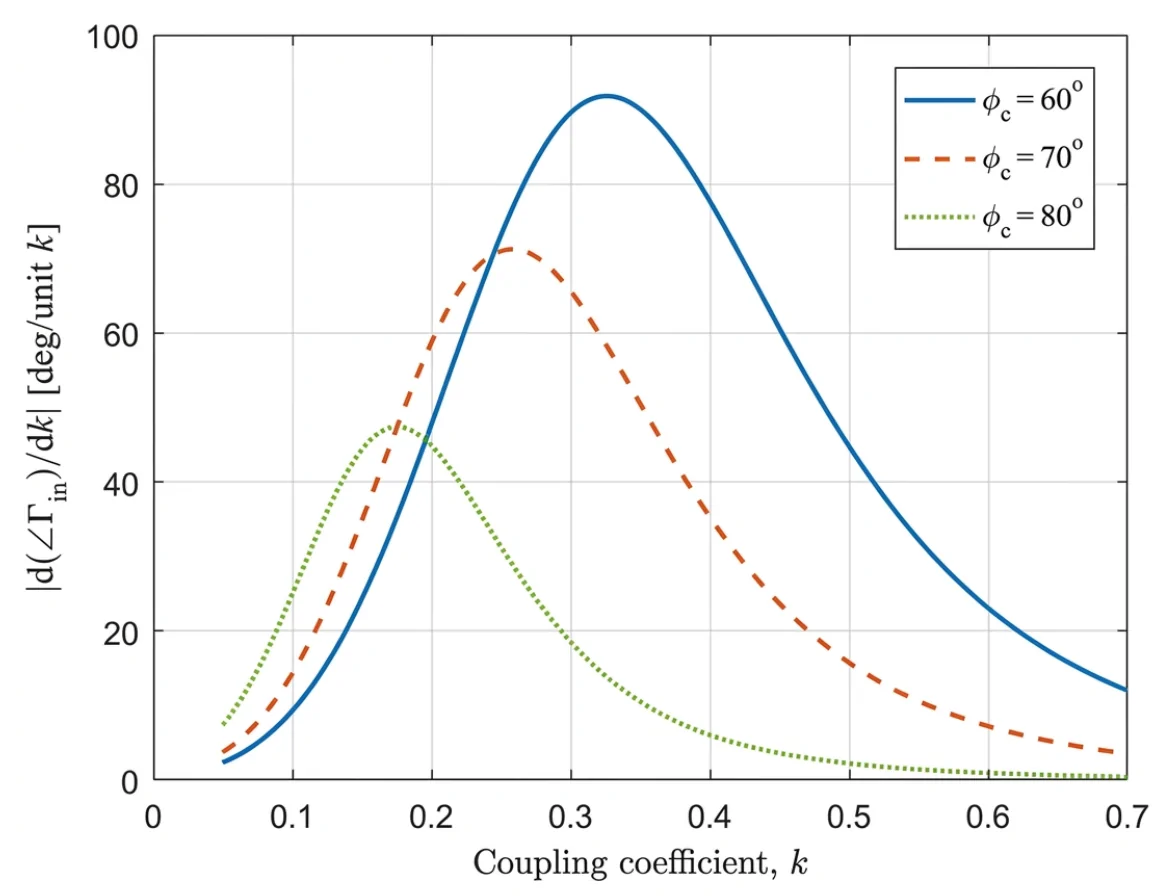
Sensing sensitivity $\left|d(∠\Gamma_{\mathrm{in}})/dk\right|$ as a function of coupling coefficient for three representative compensation angles, $\varphi_{c}=60{}^{\circ}$, 70°, and 80°. The peak sensitivity decreases from 91.77deg/unit[k] at k=0.326 ($\varphi_{c}=60{}^{\circ}$) to 71.20deg/unit[k] at k=0.258 ($\varphi_{c}=70{}^{\circ}$) and 47.38deg/unit[k] at k=0.175 ($\varphi_{c}=80{}^{\circ}$), with the peak location shifting toward weaker coupling as $\varphi_{c}$ increases. This systematic trend confirms that the sensing mechanism responds predictably to practical variations in the Chireix compensation design.

**Figure 3:**
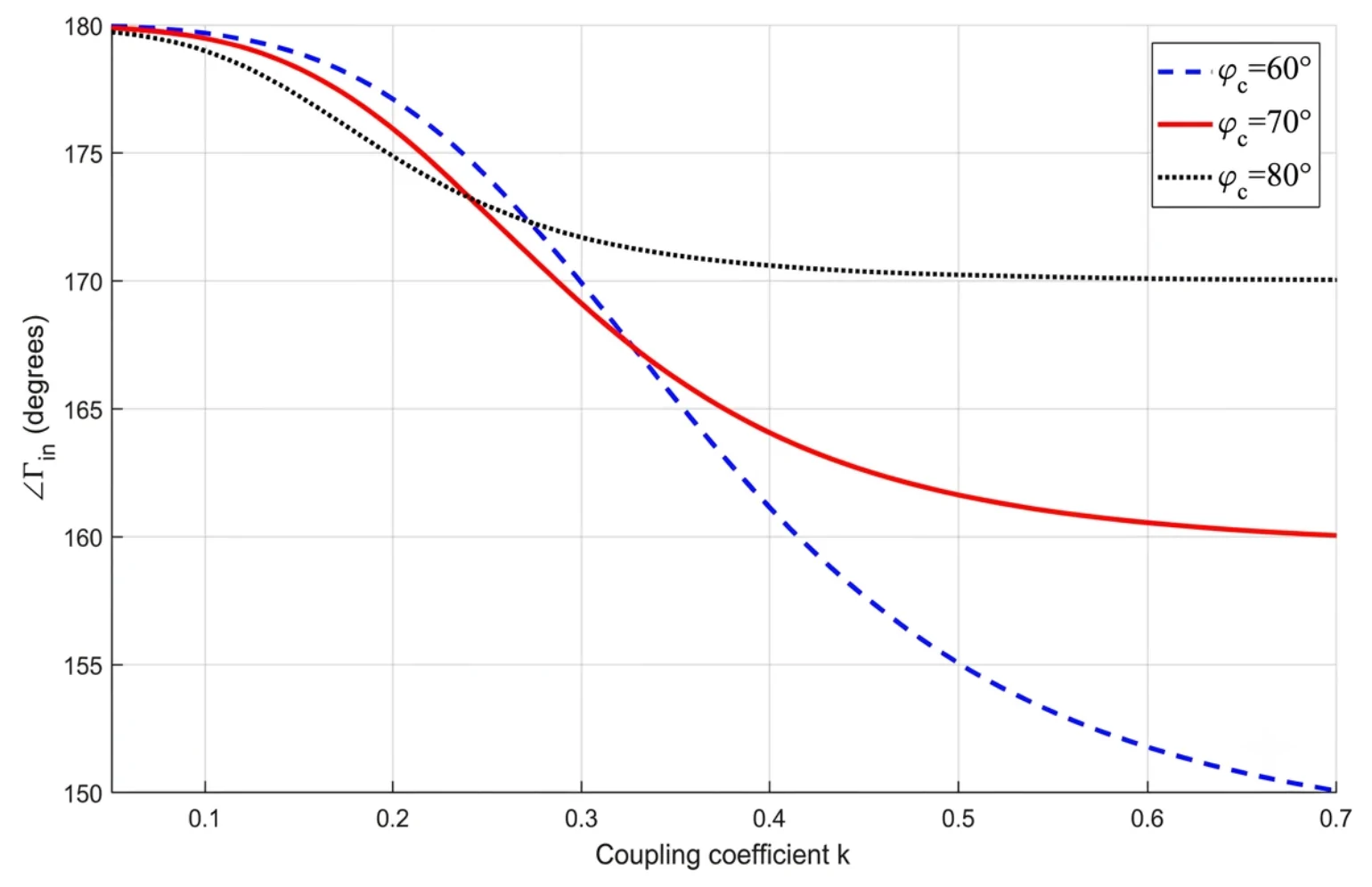
Sensitivity of the phase–coupling mapping to the Chireix compensation angle $\varphi_{c}$. Numerical monotonicity is preserved for $\varphi_{c}=60{}^{\circ}$, 70°, and 80° over k∈[0.05,0.70]. Total phase variation changes with $\varphi_{c}$: approximately 30° at $\varphi_{c}=60{}^{\circ}$, 20° at $\varphi_{c}=70{}^{\circ}$, and 10° at $\varphi_{c}=80{}^{\circ}$, confirming robustness to practical design variations in the compensation susceptance.

To assess whether the sensing principle depends critically on the specific choice $\varphi_{c}=70{}^{\circ}$, the phase–coupling relationship and its sensitivity were evaluated for $\varphi_{c}\in \left\{60{}^{\circ},70{}^{\circ},80{}^{\circ}\right\}$ while maintaining all other parameters as in Table 1. Figure 2 shows the resulting sensitivity profile $\left|d(∠\Gamma_{\mathrm{in}})/dk\right|$ for the three compensation angles, and Figure 3 confirms that the phase–coupling mapping itself remains monotonic in all three cases.

The selection of $\varphi_{c}=70{}^{\circ}$ represents a design trade-off between sensing sensitivity and phase dynamic range. Figure 2 and Figure 3 show that lower compensation angles (e.g., 60°) yield higher peak sensitivity (approximately 91.77deg/unit[k] at k=0.326) and greater total phase variation (≈30°), which in principle improve coupling resolution. Conversely, higher angles (e.g., 80°) reduce the phase span (≈10°) and sensitivity (≈47.38deg/unit[k]), limiting resolution but improving robustness against component tolerances. The 70° choice balances these competing objectives: it achieves peak sensitivity of 71.20deg/unit[k] with a phase span of 19.84° over k∈[0.05,0.70], providing adequate resolution ($3\sigma_{k}\approx 0.022$ near maximum sensitivity) while maintaining practical robustness against ±5% component variations (see the Monte Carlo analysis in Section 6.3).

## 5. Sensorless Estimation Protocol

### 5.1. Two-Step Operation

**Figure 4:**
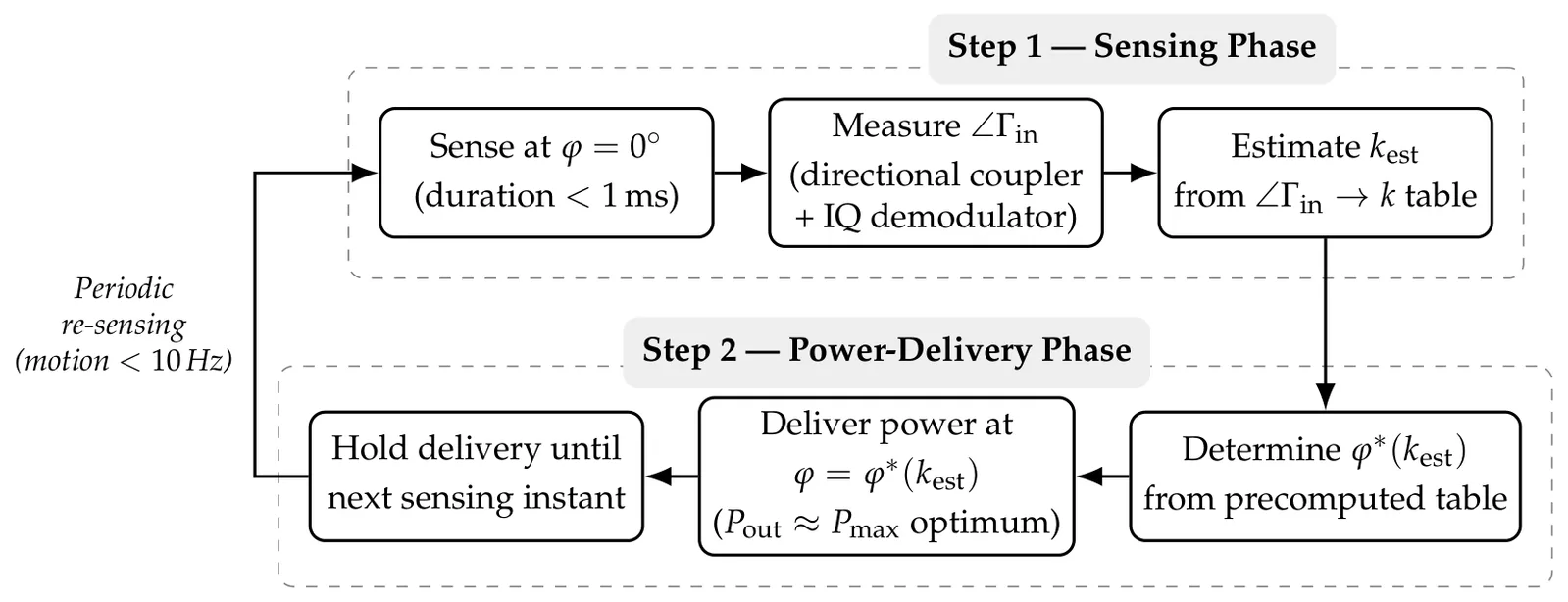
Two-step sensorless coupling-estimation and control protocol. Step 1 senses the coupling state at φ=0° using the integrated directional coupler and IQ demodulator without interrupting system power tracking. Step 2 utilizes the extracted coupling estimate $k_{\mathrm{est}}$ to update the optimal outphasing angle to $\varphi^{*}\left(k_{\mathrm{est}}\right)$ for high-efficiency adaptive power delivery, with re-sensing performed periodically relative to the human body-motion timescale.

The proposed sensorless coupling estimation exploits the numerically monotonic $∠\Gamma_{\mathrm{in}}\left(k\right)$ relationship derived in Section 4. The protocol operates in two simple steps, as illustrated in Figure 4.

**Step 1—Sensing phase (φ=0°, duration <1 ms):** Both PA branches are driven in phase (φ=0°), which corresponds to the condition of maximum combined output power ($P_{\mathrm{out}}\approx P_{\max}$). The sensing step therefore does not require the transmitter to enter a reduced-power or idle state. The phase $∠\Gamma_{\mathrm{in}}$ is measured at the combiner input port using a directional coupler and IQ demodulator [35,36]. This measurement chain requires a directional coupler, an IQ demodulator, and standard ADC/digital-signal-processing resources. While these components constitute RF measurement infrastructure, they are standard elements already present in modern digital outphasing transmitter architectures for other control and monitoring purposes, so the sensing functionality reuses existing resources rather than introducing new dedicated coupling–sensing hardware into the WPT assembly itself. As quantified in Section 6.5, a representative phase-measurement uncertainty of $\sigma_{\mathrm{phase}}\approx 0.51{}^{\circ}$ (combining IQ-demodulator and ADC quantization contributions) translates into a coupling-estimation uncertainty as low as $3\sigma_{k}=0.022$ near the point of maximum sensitivity (k≈0.25), remaining below $3\sigma_{k}=0.11$ over the weak-to-intermediate coupling range k∈[0.10,0.35]. Averaging over multiple phase samples within the sub-millisecond sensing window further improves this resolution, as detailed in Section 6.5. The sensing duration of less than 1 ms represents a negligible fraction of a typical body-motion cycle, which typically exceeds 100 ms [12,13,19].
Step 2—Power delivery phase ($\varphi =\varphi^{*}\left(k_{\mathrm{est}}\right)$): The extracted coupling estimate $k_{\mathrm{est}}$ is used to look up the corresponding optimal outphasing angle $\varphi^{*}\left(k_{\mathrm{est}}\right)$ from a precomputed table derived following the coupling-aware methodology of [33]. The outphasing angle is updated via the digital phase control already inherent to the outphasing PA architecture, and power delivery resumes at the optimal efficiency angle.

### 5.2. Sensing Lookup Table

The sensing lookup table maps $∠\Gamma_{\mathrm{in}}\to k_{\mathrm{est}}$, precomputed from Equations (7)–(10) at φ=0° using the parameters of Table 1. The full implementation uses 2000 uniformly spaced points over k∈[0.05,0.70]. Table 2 gives a representative subset.

The relatively small phase span (≈20°) results from the chosen compensation susceptance $B_{c}$, which compresses the reflection-phase dynamic range while preserving numerical monotonicity and injectivity. The total phase span of 19.84° over k∈[0.05,0.70] corresponds to a mean sensitivity of approximately 30.5deg/unit[k]. For a target estimation accuracy of Δk=0.03, the required phase resolution is about 0.03×30.5≈0.92°, which is consistent with commercially available RF phase detection capabilities [35]. The lookup table represents one practical realization of the inverse mapping $∠\Gamma_{\mathrm{in}}\to k$; equivalent implementations using direct analytical inversion or interpolation methods are equally valid.

### 5.3. Calibration Strategy

Three calibration scenarios are considered:

- **Scenario A (nominal calibration):** The lookup table is computed from nominal design parameters; no per-device calibration is required. This scenario is valid for devices within ±5% component spread, as demonstrated in Section 6.3.
- **Scenario B (ideal calibration bound):** Each device’s lookup table is constructed from its exact component values; this establishes the theoretical accuracy floor.
- **Scenario C (precision components):** With ±2% component tolerances, the nominal lookup table achieves 3σ<0.012 without per-device calibration.

## 6. Verification and Robustness Analysis of the Proposed Coupling Observability Framework

The proposed coupling observability framework is evaluated through a progressive verification strategy addressing the existence, sensitivity, robustness, and practical measurement limitations of the phase-based sensing mechanism. First, the monotonic relationship between the transmitter reflection phase and the coupling coefficient is numerically examined. Subsequently, sensitivity, parameter uncertainty, independent circuit-level verification, resonant perturbations, and phase-detection limitations are investigated. Unless otherwise stated, the results presented in Section 6.1, Section 6.2, Section 6.3, Section 6.4 and Section 6.5 were obtained by numerical evaluation of the analytical model derived in Section 4 using MATLAB R2016b. Independent circuit-level verification is presented separately in Section 6.6, where the proposed sensing principle is evaluated using S-parameter simulation performed in Keysight’s Advanced Design System (ADS) at 13.56 MHz.

### 6.1. Numerical Evaluation of the Phase–Coupling Relationship

**Figure 5:**
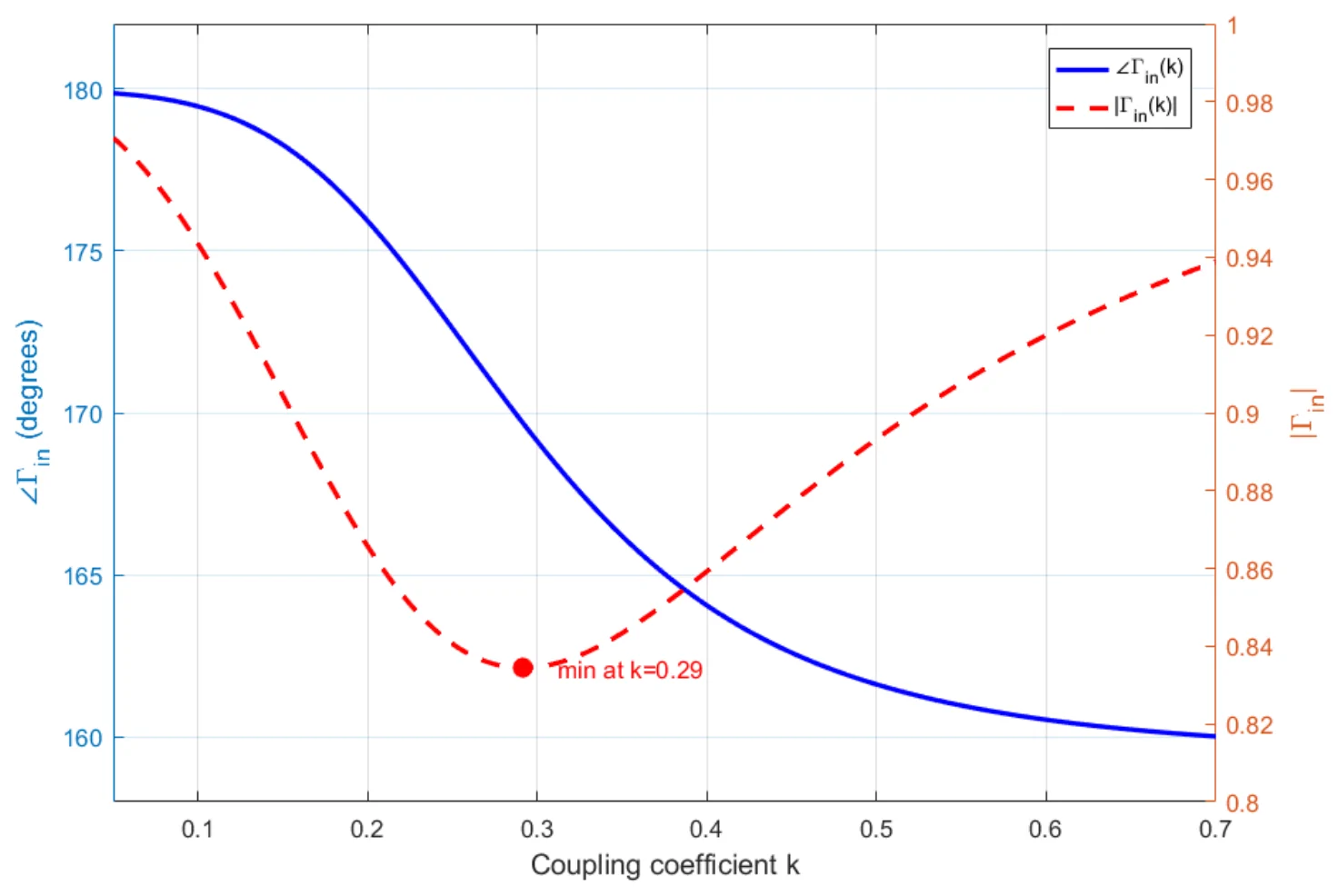
Phase and magnitude of the input reflection coefficient versus coupling coefficient at φ=0°. The phase $∠\Gamma_{\mathrm{in}}\left(k\right)$ decreases numerically monotonically from 179.86° at k=0.05 to 160.02° at k=0.70, while the magnitude $|\Gamma_{\mathrm{in}}\left(k\right)|$ exhibits a non-monotonic minimum near k≈0.29, indicating that phase is the more suitable sensing observable for sensorless coupling estimation.

The first step in evaluating the proposed coupling observability framework is to examine the dependence of the transmitter-side reflection phase on the magnetic coupling coefficient. Using the coupling-dependent equivalent impedance model described in Section 4, the input admittance, input impedance, and reflection coefficient are numerically evaluated for φ=0° over the coupling range k∈[0.05,0.70]. Figure 5 presents the resulting phase and magnitude of the input reflection coefficient as functions of the coupling coefficient.

The phase response $∠\Gamma_{\mathrm{in}}\left(k\right)$ exhibits a numerically monotonic decrease from 179.86° at k=0.05 to 160.02° at k=0.70, whereas the magnitude $|\Gamma_{\mathrm{in}}\left(k\right)|$ presents a non-monotonic behavior with a minimum near k≈0.29. These results indicate that the reflection phase provides a more suitable observable than the reflection magnitude for sensorless coupling estimation.

### 6.2. Numerical Sensitivity Analysis of the Phase Observable

The practical usefulness of the reflection phase as a coupling observable depends not only on the existence of a monotonic phase–coupling relationship but also on its sensitivity to variations in the coupling coefficient. Based on the phase response obtained in Section 6.1, the sensing sensitivity is evaluated through the numerical derivative:

(15) $$S_{k}=\left|\frac{d(∠\Gamma_{\mathrm{in}})}{dk}\right|$$

where $S_{k}$ represents the phase variation, in degrees, produced by a unit variation of the coupling coefficient.

Figure 2 presents the resulting sensitivity profile over the investigated coupling range. The sensitivity reaches a maximum value of 71.20°/unit[k] at k=0.258, indicating that the proposed observable provides its highest resolution in the weak-to-moderate coupling regime. The sensitivity remains above 3.56°/unit[k] over the full evaluated range k∈[0.05,0.70], indicating that the phase observable preserves a coupling-dependent response throughout the considered operating region.

The variation of sensitivity with coupling coefficient highlights an important design consideration: the coupling estimation accuracy is inherently dependent on the operating point. Regions of higher sensitivity provide improved resolution for a given phase measurement uncertainty, whereas reduced sensitivity increases the influence of measurement noise. The resulting uncertainty associated with realistic phase-detection errors is further quantified in Section 6.5.

### 6.3. Robustness to Parameter Variations

The practical accuracy of the proposed coupling estimation approach was evaluated through Monte Carlo analysis. A total of N=20,000 random realizations were generated for each coupling value, considering independent uniform variations in the equivalent inductive and resistive parameters of the WPT link. The resulting phase measurements were processed using the nominal phase-to-coupling lookup relationship to estimate the coupling coefficient, and the estimation error was evaluated as follows:

(16) $$\Delta k=|k_{\mathrm{est}}-k_{\mathrm{true}}|$$

Three operating scenarios were considered:

- **Scenario A:** ±5% parameter variations with nominal calibration.
- **Scenario B:** ±5% parameter variations with per-device calibration.
- **Scenario C:** ±2% parameter variations with nominal calibration.

Table 3 summarizes the resulting 3σ estimation uncertainty.

For the nominal calibration case with ±5% parameter variations (Scenario A), the proposed estimator maintains a 3σ error below 0.029 over the evaluated coupling range k∈[0.15,0.55]. This result indicates that moderate parameter deviations introduce only limited bias in the phase–coupling mapping and preserve the feasibility of sensorless coupling estimation without individual device calibration. As expected, per-device calibration significantly reduces the estimation uncertainty, while precision components further improve robustness under nominal calibration conditions.

### 6.4. Sensitivity to Resonant Detuning

**Figure 6:**
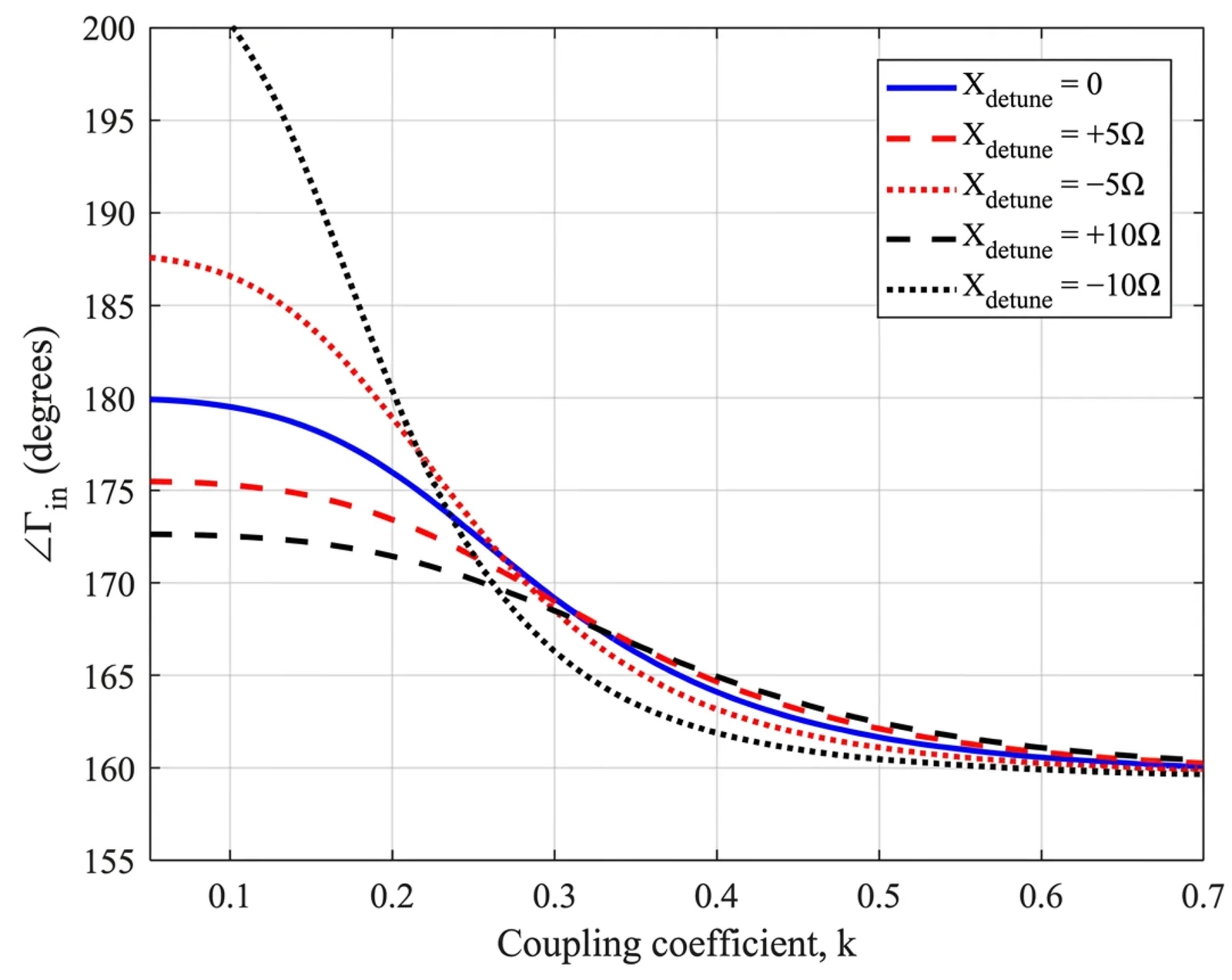
Effect of equivalent resonant perturbations on the phase–coupling mapping. The detuning reactance $X_{\mathrm{detune}}\in \left\{0,\pm 5,\pm 10\right\}\Omega$ modifies the absolute phase response while preserving numerical monotonicity over k∈[0.05,0.70]. The maximum deviation with respect to the nominal curve remains below 4.2° around mid-coupling (k≈0.30), confirming the robustness of the phase-based coupling observable against moderate equivalent reactive perturbations.

To evaluate robustness against resonant perturbations, the effective impedance model was extended by introducing an equivalent detuning reactance:

(17) $$Z_{\mathrm{eff}}\left(k,\delta \right)=Z_{\mathrm{eff}}\left(k\right)+jX_{\mathrm{detune}}$$

where $X_{\mathrm{detune}}$ represents an equivalent lumped reactive perturbation accounting for possible frequency mismatch and parasitic reactive effects. Figure 6 evaluates the influence of this perturbation on the phase–coupling relationship by numerically evaluating the analytical model of Equations (7)–(10) with the modified impedance expression for $X_{\mathrm{detune}}\in \left\{0,\pm 5,\pm 10\right\}\;\Omega$.

The introduced reactive perturbations modify the absolute phase value of $∠\Gamma_{\mathrm{in}}$, but preserve the numerical monotonicity of the phase–coupling mapping over the evaluated range k∈[0.05,0.70]. This analysis is intended to isolate the sensitivity of the proposed phase-based observable to equivalent resonant detuning within the analytical framework and it does not involve circuit-level simulation.

The results show that resonant perturbations mainly introduce a phase offset while maintaining a monotonic coupling-dependent relationship. For strong negative perturbations ($X_{\mathrm{detune}}=-10\;\Omega$), larger deviations are observed at very weak coupling (k<0.15); however, the mapping remains monotonic over the complete evaluated range. The maximum phase deviation with respect to the nominal case remains below 4.2° around mid-coupling (k≈0.30), indicating limited sensitivity of the proposed coupling observable to moderate reactive perturbations. In practical implementations, residual detuning effects can be addressed through calibration procedures that update the phase-to-coupling mapping for the specific operating conditions.

To assess robustness beyond the range shown in Figure 6, the same analytical model of Equations (7)–(10) was evaluated over an extended set of detuning values, $X_{\mathrm{detune}}\in \left\{0,\pm 5,\pm 10,\pm 15,\pm 20,\pm 25,\pm 30\right\}\;\Omega$, chosen to bracket the equivalent reactive shift associated with realistic tissue-loading conditions estimated in Section 7.5 (5–15Ω) and to probe well beyond it. For positive detuning up to +30Ω, the phase–coupling mapping remains monotonic over the complete range k∈[0.05,0.70], with the maximum deviation from the nominal curve increasing gracefully from 4.4° at $X_{\mathrm{detune}}=+5\;\Omega$ to 12.8° at $X_{\mathrm{detune}}=+30\;\Omega$.

For negative detuning, a distinct and practically relevant behavior emerges. In the range $X_{\mathrm{detune}}\in \left[-5,-15\right]\;\Omega$—which spans the tissue-loading estimate of Section 7.5—the underlying continuous phase trajectory remains monotonic, but it approaches and crosses the ±180° branch boundary within the evaluated coupling range (e.g., near k≈0.19 for $X_{\mathrm{detune}}=-5\;\Omega$, and near k≈0.17 for $X_{\mathrm{detune}}=-15\;\Omega$). Because a practical IQ-demodulator-based phase detector reports a wrapped value confined to (−180°,180°], this crossing would appear as an abrupt jump in the raw measured phase, even though the underlying physical phase varies continuously and monotonically. A lookup-table implementation that does not account for this wrap could misinterpret the coupling state near the crossing point. This effect is absent in the nominal case and under positive detuning, and it disappears again for stronger negative detuning ($X_{\mathrm{detune}}\leq -20\;\Omega$), for which the crossing occurs before k=0.05 and the entire measured phase trajectory is instead shifted to a different, still-monotonic branch (e.g., ranging from approximately 61° to 159° at $X_{\mathrm{detune}}=-20\;\Omega$, a deviation of up to 119° from the nominal mapping).

This extended analysis directly informs the practical implementation of the sensing protocol under tissue-loading conditions: because the realistic tissue-induced detuning range identified in Section 7.5 (5–15Ω) coincides with the regime in which the branch-cut crossing can occur, a robust implementation should incorporate phase-unwrapping logic—tracking phase continuity across successive sensing instants rather than relying solely on a single wrapped-phase lookup—to resolve this ambiguity near the crossing point. With this refinement, the monotonicity of the sensing principle is preserved across the full extended detuning range considered here, consistent with the robustness demonstrated for the narrower range shown in Figure 6. It is also worth noting that the effective detuning range relevant to a given wearable implementation can be substantially reduced through appropriate design of the wearable system aimed at minimizing the influence of the surrounding tissue. For example, implementing the coils on a textile substrate of sufficient thickness increases the physical separation between the coils and the body, thereby reducing the tissue-induced frequency shift estimated in Section 7.5. This reflects one of the inherent advantages of resonant inductive coupling systems, which can provide relatively robust performance in the presence of variations in the surrounding environment.

### 6.5. Phase Detection Noise, Coupling Resolution, and Error Propagation

A practical sensing implementation introduces measurement uncertainty associated with the RF acquisition chain. In the proposed architecture, the input reflection phase is obtained using a directional coupler, an IQ demodulation stage, and digital signal processing [35]. For the purpose of coupling-resolution analysis, representative phase measurement uncertainties of $\sigma_{\mathrm{IQ}}=0.5{}^{\circ}$ for the IQ demodulator and $\sigma_{\mathrm{ADC}}=0.09{}^{\circ}$ for a 12-bit equivalent ADC quantization level are considered. Assuming independent error contributions, the total phase uncertainty is given by:

(18) $$\sigma_{\mathrm{phase}}=\sqrt{\sigma_{\mathrm{IQ}}^{2}+\sigma_{\mathrm{ADC}}^{2}}\approx 0.51{}^{\circ}$$

The resulting coupling uncertainty is obtained using first-order error propagation:

(19) $$\sigma_{k}=\frac{\sigma_{\mathrm{phase}}}{\left|\frac{d(∠\Gamma_{\mathrm{in}})}{dk}\right|}$$

This relationship shows that the achievable coupling resolution depends directly on the operating point, with higher phase sensitivity providing lower coupling uncertainty for a given measurement accuracy. Table 4 summarizes the resulting $3\sigma_{k}$ coupling uncertainty across representative coupling values.

The proposed sensing approach achieves its lowest uncertainty around the maximum sensitivity region (k≈0.25), where the resulting $3\sigma_{k}$ uncertainty is 0.022. Over the weak-to-intermediate coupling range k∈[0.10,0.35], where the phase sensitivity remains relatively high, the coupling uncertainty remains below $3\sigma_{k}=0.11$.

Assuming averaging over N=10 independent phase measurements within a 100 ms observation interval, the effective phase uncertainty is reduced to:

(20) $$\sigma_{\mathrm{phase},\mathrm{eff}}=\frac{\sigma_{\mathrm{phase}}}{\sqrt{N}}\approx 0.16{}^{\circ}$$

which improves the coupling uncertainty at low-sensitivity regions. For example, at k=0.70, $3\sigma_{k}$ decreases from 0.43 to approximately 0.14. At very low sensitivity values, first-order error propagation may underestimate the uncertainty due to the nonlinear phase–coupling relationship. However, this region lies outside the primary operating regime of interest, and additional calibration or averaging strategies can further improve robustness.

Overall, these results demonstrate that the proposed phase observable exhibits the required properties for coupling-state estimation: monotonicity, sensitivity, robustness against parameter variations and resonant perturbations, and compatibility with realistic phase measurement uncertainty.

### 6.6. ADS S-Parameter Verification of the Analytical Phase–Coupling Mapping

To complement the numerical evaluation of the proposed sensing principle, an RF circuit implementation was developed using the S-parameter simulator of Keysight’s Advanced Design System (ADS). Since the sensing mechanism is derived for the ϕ=0° operating condition, where the transmitter is completely described by its linear input admittance, S-parameter analysis provides an appropriate circuit-level framework for evaluating the predicted input reflection coefficient.

The ADS implementation realizes the two-branch Chireix combiner topology using a 50 Ω input port, two compensation branches, and a common combining node. The coupling-dependent load is represented by the equivalent impedance model derived in Section 3:

(21) $$Z_{\mathrm{eff}}\left(k\right)=1+199k^{2},$$

which is assigned identically to the equivalent branch loads. The coupling coefficient was swept over k∈[0.05,0.70] with a step of 0.05, and the input reflection coefficient $S_{11}$ was extracted directly at the operating frequency of 13.56 MHz.

Unlike the harmonic-balance verification considered in the preliminary version of this work, the observable extracted in this implementation is the input reflection coefficient itself. Consequently, the ADS results correspond directly to the analytical sensing variable $\Gamma_{\mathrm{in}}$ defined in Section 4, enabling a direct point-by-point comparison between the analytical prediction and the circuit-level simulation.

**Figure 7:**
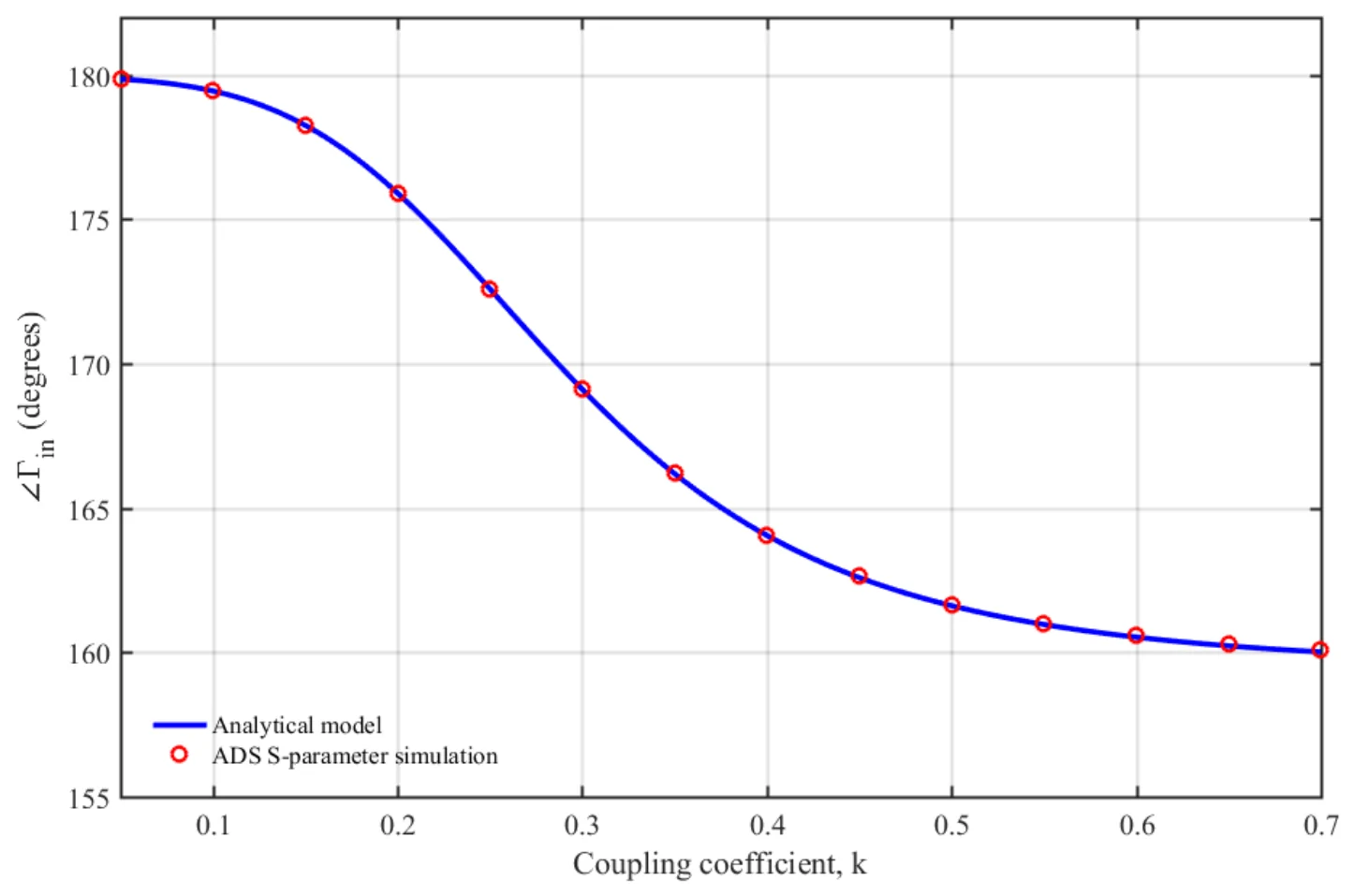
Comparison between the analytical phase–coupling mapping predicted by Equation (10) and the corresponding ADS S-parameter simulation of the Chireix circuit at 13.56 MHz. The ADS implementation realizes the two-branch Chireix topology and directly extracts the input reflection coefficient $S_{11}$. The excellent agreement between the analytical prediction and the circuit-level simulation demonstrates that the proposed phase-based sensing observable is consistently reproduced over the evaluated coupling range.

Figure 7 compares the analytical phase–coupling mapping predicted by Equation (10) with the corresponding ADS S-parameter results. Excellent agreement is observed over the entire evaluated coupling range, with the simulated points closely following the analytical prediction while preserving the unique monotonic relationship between $∠\Gamma_{\mathrm{in}}$ and *k*. The agreement demonstrates that the proposed phase-based sensing observable is consistently reproduced when the analytical impedance model is implemented within a standard RF circuit simulator using the complete Chireix branch topology.

An important clarification regarding the relationship between the analytical model and the ADS implementation is warranted. The ADS circuit-level simulation deliberately employs the same equivalent impedance model, $Z_{\mathrm{eff}}\left(k\right)=1+199k^{2}$, as the analytical derivation of Section 4. This shared model is not a validation circularity but a deliberate, layered verification choice: the purpose of the ADS simulation is not to independently re-establish the coupling-dependent impedance model itself, which follows directly from standard magnetically-coupled-resonator theory [37,38], but rather to verify that the closed-form phase–coupling relationship of Equation (10), when instantiated within a complete two-branch Chireix circuit topology (including the compensation susceptances $\pm jB_{c}$ realized as physical circuit elements rather than as idealized lumped terms), still produces the predicted input reflection coefficient. In other words, the analytical derivation isolates the sensing principle at the level of the input admittance equations, while the ADS verification isolates any errors that might arise when that admittance is realized as an actual combiner network. The two steps therefore test different potential sources of error and are not redundant with one another. A genuinely independent empirical validation of the coupling-dependent impedance model against a non-idealized, prototype WPT link—rather than against a circuit realization of the same equivalent model—remains an open task and is identified in Section 7.5 as a necessary direction for future experimental work.

### 6.7. Extended ADS Verification Under Resonant Detuning

To complement the nominal-case circuit verification of Section 6.6, the ADS implementation was extended to evaluate the analytical detuning model of Section 6.4 at the circuit level. The equivalent detuning reactance $X_{\mathrm{detune}}$ was realized as a series reactive element—a capacitor for negative $X_{\mathrm{detune}}$ and an inductor for positive $X_{\mathrm{detune}}$—added identically to both branch terminations of the two-branch Chireix topology used in Section 6.6, which is consistent with Equation (17), in which the detuning reactance modifies the shared equivalent load $Z_{\mathrm{eff}}\left(k\right)$ seen symmetrically by the combiner. At 13.56 MHz, $X_{\mathrm{detune}}=+10\;\Omega$ was realized as a series inductance of 117.4 nH, and $X_{\mathrm{detune}}=-10\;\Omega$ as a series capacitance of 1.173 nF, added to each branch termination in turn. The coupling coefficient was again swept over k∈[0.05,0.70] in steps of 0.05, matching the sweep used for the nominal case in Figure 7.

**Figure 8:**
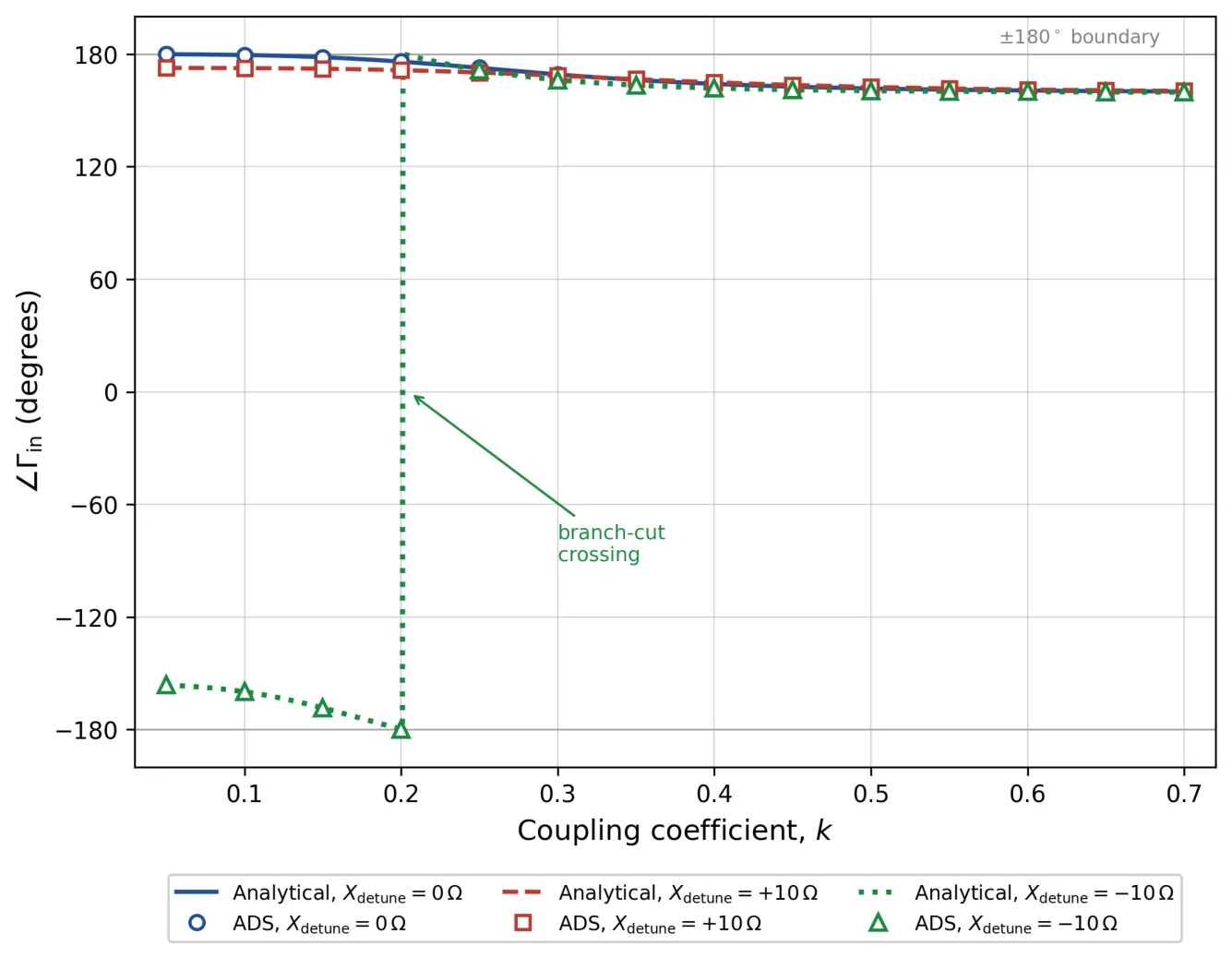
Extended ADS circuit-level verification under resonant detuning. Analytical predictions (lines) and ADS S-parameter simulation results (markers) are compared for $X_{\mathrm{detune}}\in \left\{0,+10,-10\right\}\;\Omega$, with the detuning reactance applied symmetrically to both branch terminations of the Chireix combiner. ADS and analytical results agree to within 0.10° across all cases. For $X_{\mathrm{detune}}=-10\;\Omega$, the circuit-level simulation reproduces the branch-cut crossing near k≈0.20 predicted analytically in Section 6.4, confirming that this behavior is not an artifact of the lumped-element model.

Figure 8 compares the resulting ADS $S_{11}$ phase to the analytical prediction of Equations (7)–(10) with the modified impedance $Z_{\mathrm{eff}}\left(k\right)+jX_{\mathrm{detune}}$, for $X_{\mathrm{detune}}\in \left\{0,+10,-10\right\}\;\Omega$. Agreement between ADS and the analytical model is excellent across all three cases: the maximum deviation between ADS and the analytical prediction, evaluated at all 14 simulated coupling points, is 0.05° for $X_{\mathrm{detune}}=+10\;\Omega$ and 0.10° for $X_{\mathrm{detune}}=-10\;\Omega$ (Table 5). These deviations are consistent with numerical solver precision, rather than having a physical discrepancy between the circuit-level and analytical descriptions.

Notably, the $X_{\mathrm{detune}}=-10\;\Omega$ case reproduces, at the circuit level, the branch-cut crossing phenomenon predicted analytically in Section 6.4: the raw $S_{11}$ phase decreases from −156.2° at k=0.05 to −179.8° at k=0.20, then reappears at +171.4° at k=0.25, consistent with the wrapped-phase discontinuity arising from the (−180°,180°] range of the phase computation, as discussed in Section 6.4. This circuit-level reproduction of the wrap-crossing behavior provides independent confirmation, beyond the analytical and numerical evaluation of Section 6.4, that the predicted phase behavior under moderate resonant detuning is not an artifact of the simplified lumped-element treatment but persists when the sensing principle is evaluated within a full S-parameter circuit simulation of the two-branch Chireix topology. This result reinforces the practical recommendation of Section 6.4 and Section 7.5; because the tissue-loading-relevant detuning range (5–15Ω) can produce this wrap-crossing behavior, a robust sensing implementation should incorporate phase-unwrapping logic rather than relying on a single wrapped-phase lookup.

## 7. Discussion and Practical Considerations

### 7.1. Comparison with Sensor-Based and Conventional RF Estimation Methods

Table 6 positions the proposed coupling observability framework with respect to existing sensing approaches used for wireless power transfer (WPT) systems. Unlike methods relying on additional sensing elements or dedicated measurement procedures, the proposed approach exploits the intrinsic input reflection behavior of the existing Chireix combiner, requiring no additional coupling sensor.

The main advantage of the proposed method is that coupling estimation is obtained from an RF observable already available at the transmitter interface. Therefore, the sensing functionality is integrated into the power-delivery architecture rather than added as an external subsystem.

### 7.2. Positioning Relative to Reported Coupling Estimation Techniques

Table 7 provides a quantitative positioning of the proposed approach among previously reported coupling estimation methods, including reported accuracy, coupling range, hardware overhead, and sensing duration. Because existing approaches differ significantly in terms of operating frequency, coil geometry, estimation strategy, and evaluation methodology, a fully controlled head-to-head numerical comparison is not always meaningful; the reported accuracy figures are as stated in the original references. The objective of this comparison is therefore to benchmark the proposed method against reported alternatives on the same set of practically relevant metrics.

The proposed method reports the widest validated coupling range and the shortest sensing duration among the compared alternatives. Its peak accuracy of $3\sigma_{k}=0.022$ near k≈0.25 corresponds to roughly 0.7% of the coupling value at that operating point, comparing favorably with the frequency-sweep method’s reported <8% error and the impedance-tracking method’s operating-point-dependent accuracy. Unlike the VNA-based and sensor-augmented approaches, the proposed method’s hardware overhead consists of components that can be shared with the transmitter’s existing PA control and monitoring chain, rather than dedicated external instrumentation. As with any cross-study comparison, these methods were evaluated under different operating frequencies, coil geometries, and test conditions, so the figures above should be interpreted as a positioning against reported performance rather than a controlled, apples-to-apples benchmark.
The proposed framework differs from previous approaches by introducing a new sensing perspective: the Chireix combiner is considered not only as a power-combining element, but also as an intrinsic RF interface that naturally converts coupling-dependent load variations into a highly measurable phase response.

### 7.3. Physical Interpretation of the Coupling Observability Mechanism

The coupling observability originates from the interaction between the coupling-dependent WPT load transformation and the fixed reactive compensation of the Chireix combiner. At φ=0°, the outphasing branches operate in phase, corresponding to the maximum-power operating condition. Under this condition, the combiner input admittance described in (6) reduces to a parallel combination between the coupling-dependent effective load conductance and the constant compensation susceptance.

Consequently, the combiner performs an intrinsic impedance-to-phase transformation, where variations of the magnetic coupling coefficient modify the input impedance trajectory and, therefore, the transmitter-side reflection phase. Unlike conventional impedance-monitoring approaches, where input impedance is generally treated only as an indicator of mismatch, the proposed framework intentionally exploits this impedance transformation as a sensing mechanism. This interpretation explains why the reflection phase can provide a monotonic observable of the coupling state while requiring no dedicated magnetic, capacitive, or proximity sensing hardware.

### 7.4. Practical Implementation Considerations

The proposed sensing operation requires measurement of the input reflection phase at the combiner input port. This functionality can be implemented using standard RF front-end components, such as a directional coupler, IQ demodulation stage, and digital processing unit [35].

The phase-resolution analysis presented in Section 6.5 shows that sub-degree phase accuracy is sufficient to achieve useful coupling estimation resolution. Such performance is compatible with modern RF phase-detection architectures, where phase uncertainties below the required sensing level can be achieved through appropriate calibration and averaging.

The sensing operation can be integrated into the normal WPT control sequence because the sensing condition φ=0° corresponds to the nominal maximum-power operating point. Therefore, the proposed method does not require a dedicated interruption of power transfer. A short measurement interval can be used to update the phase-to-coupling relationship while maintaining continuous energy delivery.

The additional energy consumption is mainly associated with the RF phase-detection circuitry. Using representative low-power IQ demodulation and data acquisition components, the sensing overhead remains in the milliwatt range when operated with a low duty cycle. For a transmitter delivering watt-level power, this overhead represents a small fraction of the delivered energy and is highly compatible with wearable wireless power applications.

### 7.5. Limitations and Future Extensions

The proposed sensing implementation still relies on hardware resources for phase extraction. While these components constitute RF measurement infrastructure, they are already present in modern digital outphasing transmitter architectures for other control and monitoring purposes. Compared to sensor-augmented approaches requiring dedicated magnetic field sensors, capacitive electrodes, or proximity detectors physically mounted on the coils or body, the proposed method avoids introducing new hardware elements into the WPT assembly itself. The directional coupler and demodulator operate from the transmitter interface port, leveraging the existing RF test-and-measurement capability standard in digital PA control systems.

The present study has several limitations that define the scope of the proposed framework. First, the analytical formulation assumes ideal resonance at 13.56 MHz. Practical frequency mismatch and parasitic reactive effects modify the absolute phase mapping; however, Section 6.4 demonstrates that moderate resonant perturbations preserve the monotonic coupling-dependent behavior and can be compensated through lightweight calibration.

Second, the analytical derivation assumes perfectly matched and phase-balanced PA branches. In practice, branch asymmetries introduce AM-PM distortion, gain imbalance, and finite phase skew, which prevent ideal cancellation of reactive components at φ=0° and result in a slowly varying bias term in the input admittance. In practical implementations, such branch asymmetries modify the absolute phase value of the input admittance in a manner that is structurally similar to the equivalent resonant-detuning perturbation examined in Section 6.4: both act as an additive bias on the reactive part of the input admittance rather than as a disruption of the underlying coupling-dependent conductance term. The robustness analysis of Section 6.4 (Figure 6) demonstrates that this class of additive reactive bias, evaluated over $X_{\mathrm{detune}}\in \left\{0,\pm 5,\pm 10\right\}\;\Omega$, preserves the monotonic phase–coupling mapping, with maximum deviations from the nominal curve remaining below 4.2° around mid-coupling (k≈0.30). This structural similarity provides qualitative confidence that PA branch imbalance, which introduces a bias term of the same mathematical form, would likewise be accommodated through initial calibration of the phase-to-coupling lookup table without disrupting the monotonic sensing mechanism. We emphasize, however, that no quantitative equivalence has been established between a given branch gain/phase imbalance and a corresponding equivalent detuning reactance; such a mapping would require either a detailed branch-level circuit model or direct measurement, and is identified here as a specific direction for future characterization rather than assumed in the present analysis.

Third, the sensing range has been numerically demonstrated for k∈[0.05,0.70]. This interval was selected to represent a broad range of coupling conditions relevant to practical inductive WPT applications, where coil separation, alignment variations, and application-specific geometrical constraints determine the achievable mutual coupling factor. Extension toward stronger coupling conditions requires additional investigation, particularly considering possible nonlinear effects and reduced phase sensitivity at high coupling values.

Fourth, the validation presented in this work is based on analytical derivation, numerical simulation, Monte Carlo analysis, and independent circuit-level S-parameter verification, rather than on measurements from a physical prototype. This limitation should, however, be considered alongside two complementary observations: First, at 13.56 MHz and for compact resonant WPT structures of the kind considered here, circuit-level modeling combining closed-form analytical derivation with standard RF circuit simulation (S-parameter and/or harmonic-balance analysis) is well established as a reliable predictor of measured behavior, since the dominant physical effects at this frequency—coil parasitics, dielectric loss, and near-field coupling—are well captured by lumped-element and S-parameter-based circuit models. Second, the electrical observables central to this study, namely input reflection phase and S-parameters of resonant WPT links, are measured routinely in comparable structures with excellent reported accuracy: front-end phase-detector-based monitoring has achieved relative errors below 2% [14], and frequency-sweep-based coupling estimation has achieved errors below 8% over a comparable coupling range [15]. This indicates that the measurement techniques required to validate the present method are already established in the literature, and that translating the proposed sensing principle into a measured demonstration is expected to be a tractable step once a physical prototype becomes available.

As a separate and complementary point, the authors note prior hands-on experience in fabricating and characterizing structurally similar wearable resonant coil links [7]. In that study, a controlled flexion-angle variation was used to modulate the mutual coupling between the resonant coils, analogous to the way in which coil separation and misalignment modulate *k* in the wearable WPT scenario considered here. Table 8 reproduces the measured transmission coefficient across the tested range of flexion angle from [7].

It must be emphasized that the observable measured in [7] is the transmission-coefficient magnitude $|S_{21}|$, whereas the sensing principle proposed in this work relies on the reflection-coefficient phase $∠\Gamma_{in}$. These are physically distinct quantities, obtained through different measurement chains—a scalar magnitude/power detector in the former case, versus a phase-sensitive IQ demodulator in the latter (Section 7.4)—and the reported magnitude accuracy therefore does not, by itself, establish the achievable accuracy of phase measurement. The relevance of Table 8 is accordingly narrower than a direct validation of the proposed observable: it documents that structurally similar wearable resonant coil links exhibit large, repeatable, coupling-dependent electrical responses (more than 36 dB of variation across the tested range) that can be reliably characterized using standard RF instrumentation, and that the authors have established fabrication and measurement capability for this class of device. Dedicated phase-resolved characterization—for example, using a VNA to extract $∠S_{11}$ directly, or the directional-coupler/IQ-demodulator chain described in Section 7.4—remains necessary to experimentally confirm the phase-based observable proposed here, and this is identified as a specific and distinct direction for future prototype validation.

Fifth, practical implementation introduces non-ideal effects, including branch resistance, component tolerances, and parasitic reactances. These effects mainly introduce systematic phase offsets and can be addressed through initial calibration or adaptive updating of the phase-to-coupling lookup relationship.

Sixth, the proposed observability framework relies on a lumped-element representation of the coupled WPT system. Device-level nonlinearities and electromagnetic interactions may introduce implementation-dependent deviations. Nevertheless, the fundamental monotonic relationship between coupling variation and reflection phase is expected to remain the basis of the proposed sensing mechanism.

Finally, the proposed framework has been developed for an idealized inductive WPT configuration; however, practical wearable deployments introduce additional complexities not explicitly modeled in the current analysis. Body-tissue loading modifies both the effective load resistance and introduces additional parasitic reactance, potentially altering the reflected impedance trajectory. Coil bending and deformation during body motion can modify the mutual inductance and coil series resistance. Realistic misalignment—including lateral offset, vertical separation variation, and angular tilt—introduces coupling-dependent effects beyond the idealized coaxial geometry assumed in the lumped-element model. These effects can be mitigated through appropriate system design and fabrication: coil deformation sensitivity can be reduced by appropriate optimization of the coil geometry and dimensions, informed by our prior characterization of coupling behavior in wearable coil systems under flexion and motion [6,7], and consistent with recently proposed flexible winding structures designed to preserve coupling performance under mechanical deformation [11], while the influence of biological tissues can be reduced through an appropriate implementation of the wearable system, for example by increasing the separation between the coils and the body. These wearable-specific factors primarily introduce phase offsets and modified sensitivity profiles rather than destroying the underlying monotonic phase–coupling relationship. As demonstrated in Section 6.4, the phase–coupling mapping remains monotonic across an extended detuning range bracketing this tissue-induced perturbation ($X_{\mathrm{detune}}\in \left\{0,\pm 5,\pm 10,\pm 15,\pm 20,\pm 25,\pm 30\right\}\;\Omega$); within the specific sub-range identified here (5–15Ω), however, the raw wrapped-phase observable can exhibit an apparent discontinuity at the ±180° boundary, which a practical implementation should address via the phase-unwrapping logic discussed in Section 6.4. With this refinement, practical tissue-induced detuning can be accommodated through calibration of the phase-to-coupling lookup table for the specific anatomical and geometrical configuration. A dedicated investigation of tissue-loaded WPT links at 13.56 MHz, combined with empirical characterization of realistic coil deformation under flexion, represents a logical extension of this work and would provide validation of the proposed sensing approach under clinically relevant conditions.

The tissue-detuning aspect mentioned by Reviewer 2 merits further discussion. In wearable WPT systems, biological tissue loading at the receiver side introduces frequency-dependent complex permittivity, effectively raising the dielectric loss and introducing additional capacitive reactance that detunes the resonant link. Typical human tissue at 13.56 MHz exhibits relative permittivity in the range of 40–80, introducing frequency-dependent losses that shift the resonant frequency by 0.5%–2% relative to free-space operation. This corresponds to an equivalent detuning reactance of approximately 5–15Ω at the transmitter side, falling within the range studied in Section 6.4 ($X_{\mathrm{detune}}\in \left\{0,\pm 5,\pm 10\right\}\;\Omega$). The finding that monotonicity is preserved across this detuning range (Figure 6) therefore provides confidence that tissue-induced frequency drift can be accommodated through the calibration strategy outlined in Section 5.3. A systematic measurement-based characterization of tissue-loaded WPT links, combined with in-vivo flexion studies, would provide quantitative validation of this claim and represents a prioritized direction for prototype development.

For dynamic wearable applications, the expected variation of the coupling coefficient during typical body-motion events is slow compared with the sensing interval. For motion frequencies below approximately 10 Hz, the change in coupling during a 1 ms sensing window remains negligible compared with the demonstrated estimation uncertainty. Extension toward highly dynamic scenarios, such as impact events or rapid transient motion, represents a clear direction for future investigation.

## 8. Conclusions

This paper has demonstrated that the input reflection phase $∠\Gamma_{\mathrm{in}}$ of a Chireix power combiner at φ=0° provides a numerically monotonic and locally injective mapping to the WPT coupling coefficient *k* over the wearable-relevant range k∈[0.05,0.70], enabling sensorless coupling estimation with no additional hardware. The key analytical result—that $Y_{\mathrm{in}}\left(0,k\right)=2/Z_{\mathrm{eff}}\left(k\right)-j2B_{c}$ decouples the *k*-dependent and *k*-independent components of the input admittance under ideal resonant assumptions—provides the structural foundation for the sensing principle. This result has been verified numerically over 2000 evaluation points, with the numerical derivative remaining strictly negative throughout and ranging from −71.20 to −3.56deg/unit[k], with no sign changes observed. Monotonicity has been further confirmed for compensation angles $\varphi_{c}\in \left[60{}^{\circ},80{}^{\circ}\right]$, with total phase variation ranging from 10° to 30° depending on $\varphi_{c}$.

The impedance separability condition—under which the Chireix input admittance decomposes into a coupling-dependent conductance and a fixed susceptance at φ=0°—is identified as the structural criterion enabling intrinsic coupling observability. This condition is a consequence of the Chireix outphasing topology and is generally absent in conventional single-branch resonant WPT transmitters.

The proposed two-step protocol—sense at φ=0° for less than 1 ms, and then deliver power at $\varphi^{*}\left(k\right)$ following the coupling-aware framework of [33]—is directly compatible with existing digital outphasing PA architectures, with zero hardware overhead beyond standard RF phase detection. Monte Carlo analysis confirms 3σ<0.029 under ±5% component tolerances without per-device calibration. Circuit-level ADS S-parameter simulation further supports the sensing principle by demonstrating that the input reflection coefficient at the Chireix combiner input port varies monotonically with the coupling coefficient, in direct agreement with the analytical prediction of Section 4.

Error propagation analysis shows that coupling estimation accuracy is strongly dependent on local phase sensitivity, with a best-case $3\sigma_{k}=0.022$ at k≈0.25. Temporal averaging further improves robustness at higher coupling levels where sensitivity is lower.

The proposed method transforms the Chireix combiner from a purely power-combining element into a dual-function energy transfer and sensing component, enabling real-time, low-overhead, and physically grounded coupling estimation for adaptive wearable WPT systems. Experimental validation using a wearable prototype under realistic body-motion conditions represents a natural and necessary extension. Future directions include extension to detuned resonant links, adaptation to multi-receiver WPT topologies, integration with latency-robust control frameworks, and experimental characterization of the phase–coupling mapping under real transmitter nonlinearities.

## Figures and Tables

**Table 1:** System parameters.

Parameter	Symbol	Value
Operating frequency	$f_{0}$	13.56MHz
TX/RX coil inductance	$L_{\mathrm{tx}}$, $L_{\mathrm{rx}}$	1.17μH
TX coil resistance	$R_{\mathrm{tx}}$	1Ω
Receiver load resistance	$R_{\mathrm{load}}$	50Ω
Reference impedance	$Z_{0}$	50Ω
Chireix compensation angle	$\varphi_{c}$	70°
Compensation susceptance	$B_{c}$	0.05495S
Coupling range (wearable)	*k*	0.05–0.70

**Table 2:** Phase-based sensing lookup table (φ=0°, nominal parameters).

$k_{\mathrm{est}}$	$∠\Gamma_{\mathrm{in}}$ [deg]
0.05	179.86
0.10	179.45
0.15	178.27
0.20	175.92
0.25	172.61
0.30	169.13
0.35	166.21
0.40	164.06
0.45	162.60
0.50	161.63
0.55	160.97
0.60	160.53
0.65	160.23
0.70	160.02

**Table 3:** Coupling estimation accuracy (3σ) under component tolerances. Scenario A: Nominal calibration (±5% tolerances). Scenario B: Ideal per-device calibration case. Scenario C: Precision components (±2% tolerances).

$k_{\mathrm{true}}$	Scenario A	Scenario B	Scenario C
0.15	0.0079	0.0008	0.0032
0.25	0.0132	0.0007	0.0052
0.35	0.0185	0.0008	0.0074
0.45	0.0236	0.0009	0.0095
0.55	0.0289	0.0010	0.0117

**Table 4:** Error propagation from phase uncertainty to coupling estimation ($\sigma_{\mathrm{phase}}=0.51{}^{\circ}$).

*k*	Sensitivity $\left|\frac{d(∠\Gamma_{\mathrm{in}})}{\mathrm{dk}}\right|$ [deg/Unit (*k*)]	$\sigma_{k}$	$3\sigma_{k}$	$3\sigma_{k}$ (with Averaging, $\sigma_{\mathrm{phase},\mathrm{eff}}=0.16{}^{\circ}$)
0.10	14.20	0.0359	0.108	0.036
0.25	71.20	0.0072	0.022	0.007
0.35	30.50	0.0167	0.050	0.016
0.50	15.81	0.0322	0.097	0.032
0.70	3.56	0.1433	0.430	0.135

**Table 5:** Comparison of analytical and ADS $∠\Gamma_{\mathrm{in}}$ (degrees) under resonant detuning at $X_{\mathrm{detune}}=\pm 10\;\Omega$, with the detuning reactance applied symmetrically to both branch terminations.

*k*	Analytical, −10Ω	ADS, −10Ω	Analytical, +10Ω	ADS, +10Ω
0.05	−156.29	−156.19	172.58	172.58
0.10	−159.87	−159.82	172.46	172.47
0.15	−168.49	−168.52	172.12	172.13
0.20	−179.75	−179.83	171.38	171.38
0.25	171.44	171.39	170.12	170.13
0.30	166.24	166.23	168.44	168.45
0.35	163.41	163.42	166.60	166.62
0.40	161.85	161.88	164.90	164.92
0.45	160.97	161.00	163.49	163.52
0.50	160.44	160.48	162.42	162.45
0.55	160.11	160.16	161.63	161.67
0.60	159.90	159.95	161.05	161.10
0.65	159.76	159.81	160.64	160.69
0.70	159.66	159.71	160.34	160.39

**Table 6:** Comparison with coupling and proximity estimation methods.

Method	Sensing Principle	Hardware Requirement	System Impact	Suitability for Wearables
Hall-effect [16,17]	Magnetic field measurement	Dedicated sensor + magnet	Physical integration required	Moderate
Capacitive [16,17]	Electric field variation	Electrodes + analog front-end	Environmentally sensitive	Moderate
RSSI-based [16,17]	Received power variation	RF power detector	Calibration-sensitive	Moderate
$S_{21}$-based [14,21]	Transmission coefficient	VNA or dedicated RF chain	Interrupts operation	Low
Impedance [15,18]	Input impedance variation	Directional coupler + detector	May require interruption	Moderate
**Proposed method**	Reflection phase of combiner	Reuses existing PA control RF chain (no new coil/body-mounted sensor)	Minimal overhead (<1 ms)	High

**Table 7:** Quantitative comparison of coupling estimation methods. Reported accuracy figures are as stated in the corresponding reference. Hardware overhead characterizes measurement infrastructure complexity relative to the base WPT transmitter. Sensing duration is the time required to obtain a single coupling estimate.

Method	Reported Accuracy	Coupling Range	Hardware Overhead	Sensing Duration
Freq. sweep [15]	Error <8%	k∈[0.2,0.5]	High (VNA)	>100 ms
Front-end phase [14]	Relative error <2%	Discrete *k*	Moderate (phase detector + sweep)	Sweep-dependent
Impedance tracking [18]	Varies with operating point	k∈[0.1,0.4]	Moderate (directional coupler + detector)	>10 ms
$S_{21}$ tracking [21]	Dependent on VNA calibration	Not spec.	High (VNA)	>1 s
**Proposed method**	$3\sigma_{k}=0.022$ (peak, k≈0.25); $3\sigma_{k}<0.11$ (k∈[0.10,0.35])	k∈[0.05,0.70]	Reuses existing PA control RF chain (no new coil/body-mounted sensor)	<1 ms (sensing only)

**Table 8:** Measured $|S_{21}|$ at resonance vs. flexion angle θ, reproduced from [7].

θ [deg]	2-Resonator $|S_{21}|$ [dB]	4-Resonator $|S_{21}|$ [dB]
45	−24.163	−15.979
80	−37.835	−29.294
120	−50.106	−42.495
150	−60.263	−52.729

## Data Availability

The data presented in this study, including MATLAB and Keysight ADS simulation files supporting the reported results, are available from the corresponding author upon reasonable request.

## Abbreviations

The following abbreviations are used in this manuscript:

WPT	Wireless power transfer
ISM	Industrial, scientific, and medical (band)
RF	Radio frequency
PA	Power amplifier
ADS	Advanced Design System
VNA	Vector network analyzer
IQ	In-phase/quadrature
RSSI	Received signal strength indicator
ADC	Analog-to-digital converter
